# Efficacy of Ultraviolet-C Devices for the Disinfection of Personal Protective Equipment Fabrics and N95 Respirators

**DOI:** 10.6028/jres.126.023

**Published:** 2021-08-20

**Authors:** Kumari Moothedath Chandran, Praveen C. Ramamurthy, Kawkab Kanjo, Rohan Narayan, S. Raghu Menon

**Affiliations:** 1 Indian Institute of Science, Bangalore 560012, India

**Keywords:** coronavirus disease 2019 pandemic, H1N1 viruses, NIOSH N95 respirators, personal protective equipment, test methods, ultraviolet-C disinfection, viricidal efficacy

## Abstract

Since the onset of the coronavirus disease 2019 (COVID-19) pandemic, a plethora of ultraviolet-C (UV-C) disinfection products have come to market, especially in emerging economies. UV-C–based disinfection products for mobile phones, food packaging, face masks and personal protective equipment (PPE), and other everyday objects are available in popular electronic-commerce platforms as consumer products. Product designers from multinational to startup companies began to design UV-C disinfection products but had no prior-art reference, user feedback, or validation of product efficacy, which are important stages in product design. A UV-C disinfection product cannot be assessed by most consumers for its viricidal efficacy. Many firms entered the domain of UV-C products and were unaware of the necessary validation requirements. Lack of availability and access to virology laboratories, due to lockdowns in countries, and lack of standards and certification for UV-C disinfection products limited product designers and firms in benchmarking their UV-C–based devices before market release. This work evaluates two UV-C disinfection devices for viricidal efficacy on PPE fabric and National Institute for Occupational Safety and Health (NIOSH)–certified N95 respirators through controlled experiments using the H1N1 virus, which is enveloped and is transmitted via the respiratory route similar to severe acute respiratory syndrome coronavirus 2 (SARS-CoV-2), the cause of COVID-19. The experiment also evaluated the effectiveness of chemical disinfectants along with and versus UV-C disinfection. Experiments for material selection, UV dose calculation, and UV endurance of PPE samples to be disinfected are also discussed. The outcome of this work establishes a systematic method to validate the efficacy of UV-C disinfection products. The design guidelines would benefit product designers in designing UV-C–based disinfection products.

This article was sponsored by Dianne L. Poster, Material Measurement Laboratory, and C. Cameron Miller, Physical Measurement Laboratory, National Institute of Standards and Technology (NIST). It is published in collaboration with the International Ultraviolet Association as a complement to the NIST Workshop on Ultraviolet Disinfection Technologies, 14−15 January 2020, Gaithersburg, MD. The views expressed represent those of the authors and not necessarily those of NIST.

## 1 Introduction

Coronavirus disease 2019 (COVID-19), caused by severe acute respiratory syndrome coronavirus 2 (SARS-CoV-2), is an infectious disease outbreak that started in 2019 and became a global pandemic contracted by over 94 million people, with over 2 million deaths as of January 2021 [1]. COVID-19 spreads by human-to-human transmission through droplets generated when an infected person exhales, speaks, coughs, or sneezes [2]. Several studies indicate airborne transmission via airborne infected microdroplets with acute transmission risk in indoor and enclosed environments with inadequate ventilation, prolonged exposure times, and at crowded places [3]. According to the World Health Organization (WHO), a person may also become infected by touching a contaminated surface and then touching one’s eyes, nose, or mouth before washing their hands [2]. Therefore, one of the preventive measures to avoid contracting the virus as advised by WHO is to frequently disinfect contaminated surfaces [2]. Many firms, from multinational to startup companies, quickly responded to the increased need for disinfection and launched ultraviolet (UV)–based disinfection devices for hospitals and consumer markets for disinfecting mobile telephones, laptop computers, keys, newspapers, face masks, and other everyday objects. We surveyed 99 different disinfection devices^1^1 Certain commercial equipment, instruments, or materials are identified in this paper to specify the experimental study adequately. Such identification does not imply recommendation or endorsement by the National Institute of Standards and Technology, nor does it imply that the materials or equipment identified are necessarily the best available for the purpose. from 11 countries. Devices were equipped with short-wavelength ultraviolet (UV) and ultraviolet-C (UV-C) radiation and were newly launched between March and September 2020 as advertised in the press, social media, and electronic-commerce platforms. The design of these UV-C disinfection products had either a closed-box configuration, where objects are placed inside a box, and UV-C lamps are fixed inside the box and turned on for disinfection, or an open-lamp configuration, where UV-C lamps are attached to a handheld wand or a frame on caster wheels and turned on to expose the surface to the UV-C radiation for disinfection. While 65 of the 99 UV-C disinfection products surveyed were of closed-box configuration, 34 were of open-lamp configuration (23 handheld wands, 8 space-cleaning robots, 3 sanitizer tunnels). Of the 99 products surveyed, 68 were from consumer appliance companies, 18 were from publicly funded research centers, laboratories, and universities, 6 were from engineering and technology companies, 4 were from biotechnology, healthcare, and medical device companies, and 3 were from consumer product companies.

As most companies focused on expedited market launch, product designers had to design UV-C products without prior-art reference of similar products on the market. Access to information from the traditional product development process, including market surveys, validation reports, or consumer feedback, which are important aspects in a product design process, was also difficult during lockdowns, during which governments restricted human movement and commerce. Most firms and organizations who launched new UV-C disinfection devices during March 2020 to September 2020 were found to be from a different product segment background and newly entered in the UV-C disinfection product segment [4]. Therefore, product designers and firms were also limited by lack of design guidelines, product safety requirements, and efficacy, standards, and certifications for UV-C–based disinfectant products with which to benchmark their UV-C–based product before market release. This means consumer feedback was restricted to the industrial design, user friendliness, and affordability. Moreover, the main functionality of a UV-C disinfectant product is its viricidal efficacy, which cannot be assessed by consumers through feedback. Due to lockdowns on movement and commerce imposed in various countries by governments to prevent the spread of virus, availability and access to virology laboratories too were limited, as these laboratories were also conducting diagnosis functions or higher priority research on vaccines.

We designed and developed a UV-C disinfection device during a nationwide lockdown in India from mid-March 2020 to May 2020. The design requirement was to design a device for disinfecting personal protective equipment (PPE), which includes coveralls and National Institute for Occupational Safety and Health (NIOSH)–certified N95 respirators made of fabric, where N95 indicates a capability to filter 95% of airborne particles.^2^2 “N95” is a filter class designation of the U.S. National Institute of Occupational Safety and Health (NIOSH). It is applied to respirators that are at least 95% efficient at filtering NaCl aerosols with particle sizes of mean diameter 75 nm ± 20 nm (NIOSH Procedure No. TEB-APR-STP-0059, 13 December 2019). As PPE was in dire shortage in India in the beginning of the pandemic, the purpose was to extend the effective life from single use for noncritical care use cases at institutional buildings like hospitals and clinics. There was no prior study on the viricidal efficacy of UV-C exposure on PPE fabrics, and that was the motivation for this work. We evaluated two different UV-C disinfection unit configurations and evaluated their viricidal efficacy through controlled experiments to arrive at the appropriate design requirements for the design of a new UV-C disinfection device for PPEs. This work discusses all aspects of the product design process, from material selection of the device to UV-C lamp selection and dose calculation, endurance of PPE fabrics to UV-C radiation, and viricidal efficacy of chemical disinfectants on PPEs along with and versus UV-C disinfection. The outcome of this work establishes potential evaluation approaches that can be used to evaluate a UV-C disinfection device and provides design guidelines for product designers designing UV-C disinfection products.

## 2 Overview of Product Design Process

The newly designed UV-C disinfecting device was for the disinfection of PPE coveralls and N95 respirators. The UV spectrum lies from 200 nm to 400 nm and has three regions: UV-A: 320 nm to 400 nm, UV-B: 280 nm to 320 nm, and UV-C: 200 nm to 280 nm [5]. A PPE kit contains a coverall, an N95 respirator, gloves, and face and eye protection shields. As the nationwide lockdowns delayed the supply chain of PPEs to hospitals, there was a need for healthcare staff to extend the life of N95 respirators and PPE coveralls for non–intensive care unit use. As the government of India encouraged production of PPEs among fabric garment manufacturers on a war-time basis, we procured sample PPE kits and fabric samples from manufacturers to check their suitability for UV-C disinfection. The product design process was started by a team of designers for rapid development. Sourcing, manufacturability, and availability of laboratories for testing during lockdown were the major constraints in the design process. A systematic product design approach was followed [6, 7], and the steps carried out at each design stage are explained below and summarized in Table 1.

**Fig. 1:**
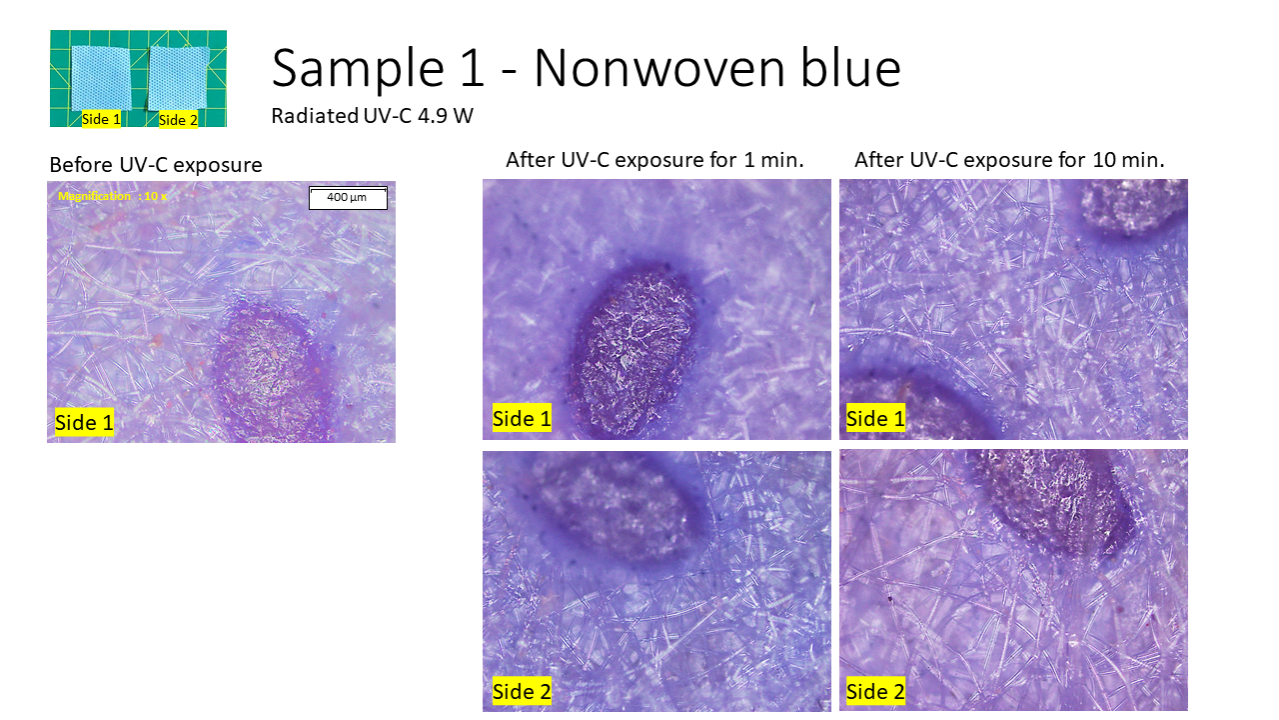
Endurance test #1 UV-C, Sample 1: PPE coverall fabric (nonwoven) showing no changes.

**Fig. 2:**
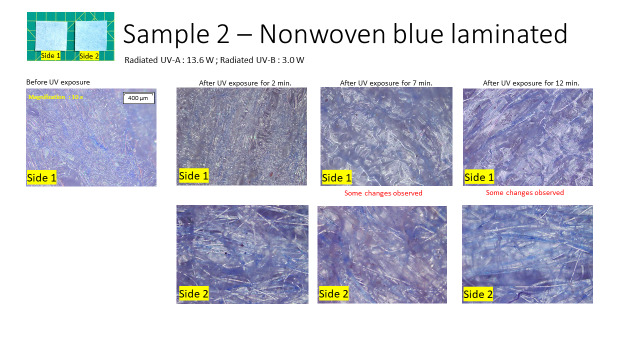
Endurance test #2 UV, Sample 2: PPE coverall fabric (nonwoven, laminated), showing changes in the laminated side.

1. Planning and clarification of task or feasibility:
  a. Different types of PPE coverall fabric samples were sourced from manufacturers of PPEs. These were checked under an Olympus BX53M upright light microscope to identify the fabric type. PPE coverall fabrics are mostly nonwoven materials. Some fabrics are laminated with a plastic lining. Figures 1 and 2 show microscopic images of nonwoven fabrics with and without lamination.
  b. The second step was to check if the PPE samples could endure UV radiation without any changes to their structural properties. If changes were observed, the product design process could not be carried forward, because the main requirement of PPE reuse would be infeasible. For this purpose, we carried out the UV endurance test explained in Sec. 3.1.
2. Conceptual design:
  a. An important design calculation in UV disinfection is the UV dose calculation. The UV dose based on the literature was used to arrive at the intensity and UV dose required to disinfect the virus as described in Sec. 3.
  b. Selecting a suitable UV-C lamp was the next step. The sourcing team obtained UV-C lamps that were available locally. Although the product catalogue of lamps provides information on the dominant wavelength and wattage, the lamps had to be tested to check the UV spectrum, intensity, time span of the wavelength emitted, and the distance up to which it is detected.
  c. After lamp selection, different concepts were generated for disinfecting N95 respirators and PPE coveralls.
  d. To evaluate the concepts and test the selected lamp’s viricidal efficacy before going forward to the next design stage, Experiment #1 in Sec. 3.3 was done.
3. Embodiment design: The design team identified available material and manufacturing facilities. Once these were confirmed, the concept was further developed.
4. Detail design: The design of controls and detailed drawings were developed.
5. Design for manufacture: The material was procured, and prototyping was carried out at this stage. Controls were assembled on site. The finished prototype was tested for safety and viricidal efficacy. Experiments #2–7 explain this step. Before the release of the product, the safety of the product against a UV leak through product enclosure or with an accidental opening was tested. Experiment #8 in Sec. 3.5.2 was done for this purpose.

**Table 1:** Design stages of UV-C device identification, acquisition, and characterization and tests required at each stage.

Design Stage	Steps	Tests Required
Planning and clarification of task or feasibility	Source PPE samples	Verifying fabric type of samples under microscope, whether laminated or not and woven or nonwoven
	Check PPE fabric endurance during exposure to UV radiation	UV-C endurance test on PPE samples intended to be disinfected
	Conduct literature study of UV product standards and test procedures	
Conceptual design	Calculate UV dose	See Sec. 3
	Select and source UV lamp devices	UV lamp characterization: UV-C intensity, wavelength, and UV dose measurement at a specified distance (maximum distance from source with required UV dose)
	Design concepts for the UV device	
	Evaluate concepts	Viricidal efficacy of the chosen lamp/product
Embodiment design	Finalize overall dimensions, parts, and design of controls	UV-C reflectivity of internal material in a device
Detail design	Finalize final dimensions, component selection, tolerances of parts, design of controls Create detailed drawings for manufacturing and assembly	
Design for manufacture	Complete sourcing and prototyping	
	Generate certifications and product safety guidelines	UV-C product certification: product UV-C properties—UV-C intensity, wavelength, and UV dose at boundary points of the product Product safety test during use (UV leak and human exposure)

### 2.1 UV-C Product Physical Standards and Test Procedures

As outlined in Table 1, we faced the need to carry out tests for the following five aspects:

1. UV-C endurance test on PPE samples to be disinfected;
2. UV-C lamp characterization: UV-C intensity, wavelength, and maximum distance from UV-C source with required UV dose;
3. viricidal efficacy of the chosen lamp (*i.e.*, product);
4. UV-C reflectivity of materials selected; and
5. UV-C product certification: UV-C properties of the final product—UV-C intensity, wavelength, and UV dose at boundary points of the product; product safety test during use (UV leak and human exposure).

For these five tests, we searched for existing standards and test procedures on UV lamp use and disinfection that could be adopted for the tests. Table 2 lists the standards found, and their suitability is discussed below.

To check the suitability of UV-C disinfection of PPEs, it is essential to check the endurance of PPE fabrics subjected to UV-C exposure, which indicates whether the fabric properties are affected by UV-C and the number of cycles of disinfection that can be carried out without damaging the fabric. Existing standards and test methods for UV use on fabric are for sun-protective clothing, which primarily look for transmittance of UV-B wavelengths of 280 nm and above. Australian/New Zealand Standard AS/NZS 4399:1996 Sun protective clothing—Evaluation and classification [8], American Association of Textile Chemists and Colorists AATCC 183:2020 [9], American Society for Testing and Materials ASTM D6544 [10], and ASTM D6603 [11] in the United States, and European Standard EN 13758-2 [12] in Europe are the standards in this area. The UV protection factor (UPF) value is a rating system used for fabrics that characterizes a time factor for the protection of Caucasian skin compared to exposure without any protection [13]. These standards provide information on the relative capability of textiles and apparel to provide protection against solar UV radiation (UV-A and UV-B). While there are no peer-reviewed studies available on the effect of UV-C on fabric properties such as structure and strength, solar UV effects on cellulose cotton have been shown to cause increased color, yellowness, and carboxyl content and decreased dye adsorption and breaking strength [14]. The relationship between a fabric’s physical characteristics and UV radiation transmission has also been widely discussed in the literature [15].

The International Organization for Standardization (ISO) 15727:2020 (Measurement of the output of a UV-C lamp) [5] is a relatively new test (January 2020), for which the ISO test centers in Bangalore were not equipped. The ASTM E1837–96 (Standard test method to determine efficacy of disinfection processes for reusable medical devices) is for disinfecting medical instruments designed for reuse, using an established disinfection method [16]. This method could not be used because we were attempting to disinfect PPEs that were not designed originally for reuse. However, this method could be used for the UV disinfection of devices used daily or household items, such as phones, keys, or packaging materials, which are not single-use products. Moreover, there were insufficient data on the efficacy of UV-C on SARS-CoV-2 virus at the time of writing of this article. Therefore, UV-C is not yet an established disinfectant for COVID-19. As described by the U.S. Food and Drug Administration’s public website, there are limited published data about the wavelength, dose, and duration of UV-C radiation required to inactivate the SARS-CoV-2 virus [17].

The ISO 15858:2016 (Safety information—Permissible human exposure) standard is for testing UV-C lamps in heating, ventilation, and air conditioning (HVAC) systems with air flow [18]. However, the UV-C system designed in our work was a closed device with no ventilation. The American National Standards Institute/Illuminating Engineering Society of North America (ANSI/IES) RP-27 [19] and International Electrotechnical Commission (IEC) 62471 [20] apply to practices for the safe use of lighting systems in photobiology applications, which was not in scope of the product development, as UV-C lamps readily available in the market were procured. Since the UV disinfection product segment is new, the ISO test centers in Bangalore have no established UV-C testing capabilities.

Due to the lack of clear test procedures or laboratories available, we carried out tests at different university laboratories at the Indian Institute of Science, Bangalore, and devised new experimental methods to test the five aspects listed earlier in the text and described in Table 1.

**Table 2:** UV standards and test procedures.

Test Type	Standard/Test Name	Description	Application
Object property	AS/NZS 4399:1996 Sun protective clothing— Evaluation and classification [8]	This standard provides information to the consumer on the relative capability of textiles and articles of personal apparel to provide protection against solar UV radiation in order to assist consumers in the selection of those items that best suit their need for sun protection.	Sun-protection textiles and apparel
Object property	AATCC 183 2020 Standard practice for preparation of textiles prior to ultraviolet (UV) transmission testing [9]	This test method provides a method to determine the UV radiation transmitted or blocked by textile fabrics.	Textile fabrics intended to be used for UV protection in dry or wet state
Object property	ASTM D6544 2012 Standard practice for preparation of textiles prior to ultraviolet (UV) transmission testing [10]	This practice covers standardized exposures to laundering, simulated sunlight, and chlorinated pool water to which cloth, labeled as ultraviolet-(UV) protective, must be exposed prior to testing for UV transmission. This practice leads to measurement of the residual level of UV protection in fabrics or garments labeled as sun- or UV-protective after exposure to conditions that relate to about two years of seasonal use.	To be used in support of a label statement regarding UV protection of fabrics
Object property	ASTM D6603 2019 Standard specification for labeling of UV-protective textiles [11]	This standard describes labeling requirements for textile products intended for the protection of humans from UV-A and UV-B radiation.	Specifies terminology to be used in the labeling of UV-protective textiles
Object property	EN 13758 2006 The European standard for sun‐protective clothing [12]	This European standard provides test methods for spectrophotometric measurements for textile materials and classification and marking of apparel textiles.	Testing and labeling of UV‐protective summer clothes
UV-C Lamp output	ISO 15727:2020 Measurement of the output of a UV-C lamp (2020-01) [5]	This international standard specifies the measurement of the output of a UV-C lamp, types of UV-C lamps, lamp ballast, and safety issues.	Linear UV-C disinfection lamps, UV-C lamps installed in heating, ventilation, and air conditioning (HVAC) systems
Disinfection efficacy	ASTM E1837–96 (2014) Standard test method to determine efficacy of disinfection processes for reusable medical devices (simulated use test) [16]	This standard test method provides a reproducible procedure to verify the effectiveness of a previously validated disinfectant or disinfection procedure for reusable medical instruments and devices by simulating use situations.	Can be used to verify claims of disinfection of recesses, hinged sites, lumina, or other difficult-to-reprocess areas of reusable medical devices and instruments Can be used to document the contribution of each element of the reprocessing cycle for reusable medical devices and instruments
UV-C lamp device safety	ISO 15858:2016 Safety information—Permissible human exposure [18]	This international standard specifies minimum human safety requirements for the use of UV-C lamp devices.	In-duct UV-C systems Upper-air in-room UV-C systems Portable in-room disinfection UV-C devices Any other UV-C devices that may cause UV-C exposure to humans Not applicable to UV-C products used for water disinfection
UV-C lamp safety	ANSI/IES RP-27 Recommended practice for photobiological safety for lamps and lamp systems [19]	This recommended practice is for the evaluation and control of optical radiation hazards from all electrically powered sources of optical radiation that emit in the wavelength range from 200 nm through 3000 nm.	Suitable for UV-C lamps with wavelength range of 254 nm
UV-C lamp safety	IEC 62471 Photobiological safety of lamps and lamp systems [20]	This international standard gives guidance for evaluating the photobiological safety of lamps and lamp systems including luminaires. Specifically, it specifies the exposure limits, reference measurement technique, and classification scheme for the evaluation and control of photobiological hazards from all electrically powered incoherent broadband sources of optical radiation, including light-emitting diodes (LEDs) but excluding lasers, in the wavelength range from 200 nm through 3000 nm.	Suitable for UV-C lamps with wavelength range of 254 nm

## 3 Experimentation and Test Methods

In this section, we present the test methods devised and the experimental protocols used to evaluate the five tests described in Sec. 2.

### 3.1 Test 1: UV Endurance Test on PPE Fabrics

We carried out two tests using two UV radiation sources to check the endurance of PPE fabrics subjected to UV radiation, which are explained below:

1. Endurance test #1 UV-C: Three PPE coverall fabric samples were exposed to UV-C for 1 min and 10 min. The samples were then observed under an Olympus BX53M upright light microscope. The UV-C source was a Philips TUV 15W UV-C Lamp (radiated UV-C 4.9 W). The time stamps were chosen considering 1 min as one disinfection cycle time, and 10 min as 10 disinfection cycles at maximum before discarding a PPE.

For endurance test #1 using UV-C, no change was observed for all three samples after 10 min of exposure. Figure 1 shows results of sample 1. Samples 2 and 3 are shown in Appendix A.1.

2. Endurance test #2 UV: Eight PPE coverall fabric samples were each exposed to UV for 2 min, 7 min, and 12 min. The samples were then observed under an Olympus BX53M light microscope each time. The UV source was an Osram Ultra Vitalux 300 W lamp (UV-A 13.6 W; UV-B 3 W).

**Fig. A.2.1:**
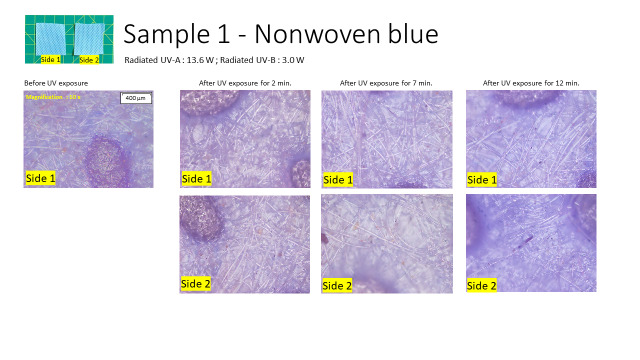
Endurance test #2 UV, sample 1: PPE coverall fabric (nonwoven, laminated) showing no changes as examined using an Olympus BX53M upright light microscope.

For endurance test #2 UV, we chose a higher wattage lamp and higher wavelength UV to check for fabric behavior on exposure to UV-A and UV-B radiation. Changes were observed on seven out of the eight samples. Four selected results are presented in this paper. Figure 2 shows the results of sample 2, a laminated nonwoven fabric. The remaining three samples are shown in Appendix A.2. The changes observed were on the laminated side in both laminated woven and nonwoven samples that showed changes in the structure of the laminate appearance under the light microscope (see Figs A.2.1 and A.2.2.). Thinning was observed on nonwoven fabric fibers at 7 min of UV exposure, where the cross-sectional area of the fibers reduced and blister-like bulging of the fibers at certain points was noticed. The laminated side looked melted in these samples. A smell was observed in two samples. These results indicate that the PPE fabric examined in this study undergoes structural changes upon exposure to UV. The reasons for the changes could be due to UV-A or UV-B radiation or any slight increase in the heat generated due to the high-wattage lamp, and this needs to be investigated further.

The above test method followed a visual analysis to determine the changes. Both the endurance tests did not assess the tensile strength of the fabric. This could be performed using ISO 13934-1:2013(en) Textiles—Tensile properties of fabrics [21]. The drawback here is that ISO 13934-1:2013 is intended for woven fabrics, whereas most PPE coveralls are made of nonwoven fabrics, which have no distinct direction of maximum strength in the fabric due to the random nature of fiber structure. For nonwoven fabric, a test for tensile strength can be performed on fabric swatches cut from different portions of the sample fabric PPE garment (from each side and seams), and an average tensile strength can then be calculated. The samples can then be exposed to UV-C and checked again similarly for average tensile strength. By repeating tests for different UV exposure time stamps, the maximum UV exposure duration that a nonwoven PPE fabric can endure can be obtained. This method needs to be tested and validated.

### 3.2 UV Lamp Characterization

In order to select a UV-C lamp, we tested the UV-C wavelength and intensity and calculated the dose it emitted at 15 cm for several lamps procured. UV radiation affects a virus by introducing irreversible mutations in the viral genome, thereby rendering them noninfectious [22, 23]. The stability of influenza and SARS-CoV-2 has been reported to be an average of 48 h to 72 h on various surfaces [24, 25]. The results may vary as per the methodology used for analyzing virus infectivity (*i.e.*, quantifying infectious virus particles versus detection of virus genetic material by polymerase chain reaction [PCR] amplification).

UV dose is expressed in millijoules per square centimeter (mJ/cm^2^) [26]. It is a product of UV irradiance and specific exposure time on a given microorganism or surface [26]. The UV dose for 90% inactivation is given as D90 values [27]. The results from prior studies on UV susceptibility of coronaviruses have D90 values in a range of 7 J/m^2^ to 2410 J/m^2^, with 237 J/m^2^ as the average of all studies [27]. UV susceptibility is the extent to which a microorganism is sensitive to UV radiation or how easily it can be inactivated by UV irradiation [26]. Kowalski *et al.* [27] reported a mean D90 value as 47 J/m^2^ for a range of coronaviruses, excluding outliers from various studies, but this was viewed as conservative, as the data set included two SARS-CoV-2 studies with an average D90 value of 27 J/m^2^.

According to ISO 15714:2019 [26], the UV dose can be calculated for a device with evenly distributed UV irradiation and air flow. It also says that for most real in-duct UV germicidal irradiation (UVGI) devices, the UV dose to every single microbe is different [26]. Therefore, we checked the wavelength and intensity of UV-C lamps using an Ocean Optics USB4000 spectrometer and calculated the UV dose at 15 cm from the radiation source. Table 3 shows a compilation of the lamps procured and tested. The maximum intensity value the spectrometer reported was 64 027 counts, so values above that are given as >64 027, and corresponding UV dose values are given as >58.61 J/m^2^ in Table 3. The Osram Puritec germicidal lamp with 4.9 W radiated power was selected for the viricidal efficacy studies based on the results in Table 3 and its dimensional suitability.

**Table 3:** UV lamp intensity testing and dose data.

Lamp Manufacturer	Radiated UV-C Power as in Product Data Sheet (W)	Wavelength as in Product Data Sheet (nm)			Wavelength Detected in Spectrometer (nm)	Intensity Detected in Spectrometer (counts)	254 nm UV Dose Calculated at 15 cm Distance (J/m^2^)	
Philips UV-C 15 W (45.72 cm)	4.9	245–255	250–275			>64 027.00	>58.61	
Philips TUV Mini 11 W (22.6 cm)	2.6	250–275	250–275			>64 027.00	>58.61	
Osram Puritec 15 W (43.8 cm)	4.9	254 dominant	250–280			>64 027.00	>58.61	
A commercial UV LED	1	254		250–280		>64 027.00		> 8.24

### 3.3 Test for Viricidal Efficacy

**Fig. 3:**
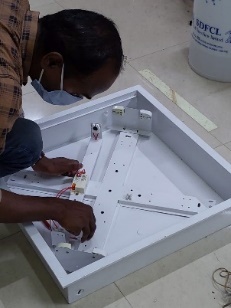

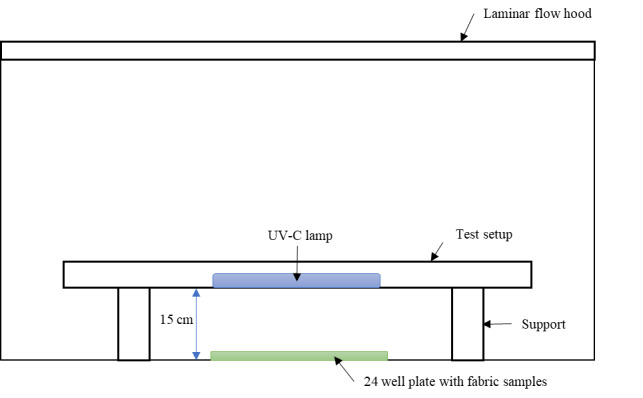
(Left) Test setup photo and (right) schematic diagram for viricidal efficacy experiment #1 UV-C.

After selecting the lamp, concepts were generated for the UV-C device, which included both open-configuration and closed-configuration designs. A test setup with a size of (62 × 62 × 20) cm was built using the selected lamp (see Fig. 3). It had powder-coated sheet metal casing covering all sides except the side where samples for disinfection would be kept. This open-configuration test setup was then checked for its viricidal efficacy and to verify the exposure time as per theoretical calculations. As Kowalski *et al*. [27] showed that 47 J/m^2^ was a conservative D90 value for all coronaviruses so far reported; as a factor of safety, 100 J/m^2^ was considered in the design calculations.

*UV-C Dose* (W s/m^2^) *= UV Irradiance* (W/m^2^) *× Exposure* (s), (1)

where

*UV Irradiance* (W/m^2^) *= Radiated power* (W)*/ Area of lamp* (m^2^) . (2)

Therefore, exposure time is equal to (100 × 2π × 0.013 × 0.438)/4.9 = 0.73 s, where the dose required is 100 J/m^2^, radiated power is 4.9 W, and UV radiation source (cylindrical tube) surface area is 2π*rl*, where tube radius *r* is 0.013 m, and length *l* is 0.438 m.

Coronaviruses are enveloped, single-stranded, positive-sense ribonucleic acid (RNA) viruses of the family *Coronaviridae* that contain spike glycoproteins protruding from their surface, giving the appearance of “crowns” [28]. The ongoing COVID-19 pandemic, which is caused by SARS-CoV-2, is related to this family of viruses [29]. In this work, the viricidal efficacy testing was performed using H1N1 viruses. Similar to SARS-CoV-2, these viruses are enveloped and are transmitted via the respiratory route. The viricidal efficacy of UV-C on H1N1 is D90 = 13 J/m^2^ for 222 nm UV-C as per the literature [30]. From Eq. (1), assuming a dose of 13 J/m^2^ with a safety margin at 30 J/m^2^, the exposure time required would be 1.07 s for disinfecting the H1N1 virus.

#### 3.3.1 Experiment #1 UV-C: Viricidal Efficacy of UV-C Test Setup (Open Configuration)

In experiment #1 UV-C, the viricidal efficacy test was performed using the A/California/04/2009(H1N1) virus strain that was genetically modified to express green fluorescent protein (GFP). The influenza A virus–GFP reporter–based fluorescence assay (FA) [31] and plaque assays can both be used for quantification of infectious virus particles. In the former, GFP can be expressed only during active virus replication, and hence the number of GFP-positive cells is a direct indication of infectious virus particles. This technique is widely used in virology.

The virus was grown in Madin-Darby Canine Kidney (MDCK) cells and titrated by plaque assay in the same cell line. The virus was originally isolated from a 10-year-old male on 01 April 2009 in California, USA, during the 2009 Influenza A H1N1 virus pandemic. The virus infection experiment was performed in A549 (human lung alveolar adenocarcinoma cells).

**Fig. 4:**
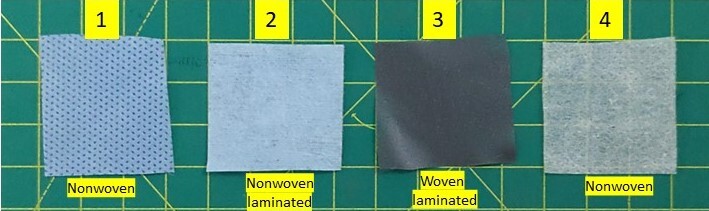
The four PPE fabric samples used for viricidal efficacy in experiment #1 UV-C.

**Fig. 5:**
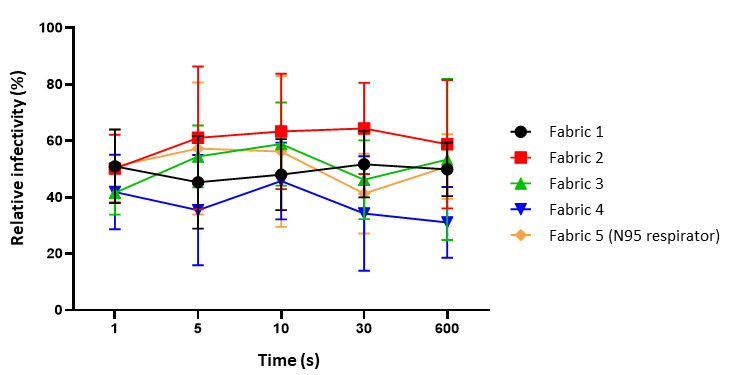
Viricidal efficacy of UV-C test setup in an open configuration. The *y* axis is the percentage of infected cells relative to the untreated virus control. The *x* axis is the time of treatment. The error bars represent the mean standard deviation of the replicates (*n* = 3).

Four PPE coverall fabrics samples—nonwoven (2), nonwoven laminated (1), woven laminated (1)—and an N95 respirator were used as test samples (see Fig. 4). The fabrics were cut into 0.5 cm × 0.5 cm pieces, placed in a 24 well cell culture plate in triplicate, and UV irradiated for 30 min before beginning the assay. A 50 µL (1.5 × 10^7^ plaque-forming units or PFU) volume of virus was placed over the fabrics suspended in OptiMEM (reduced serum medium) and exposed to UV-C in the open-configuration UV device for 1 s, 5 s, 10 s, 30 s, and 10 min. The samples were kept at 15 cm from the radiation source (see Fig. 5). The virus from each sample triplicate was then pooled together and resuspended in 300 µL OptiMEM. Infection was done in triplicate by adding 100 µL of the pooled virus suspension per well in a 24 well cell culture plate containing 0.2 million A549 cells per well grown on 12 mm glass coverslips. The cells were incubated at 37 °C for 60 min to allow virus adsorption and then topped up with 400 µL OptiMEM in each well. After 9 h of incubation, cells were fixed, nuclei were labeled using DAPI (4′,6-diamidino-2-phenylindole), and images were captured using an EVOS M5000 imaging system. DAPI is a fluorescent stain that binds to deoxyribonucleic acid (DNA) and is used to label the cell nuclei. The medium in the wells was aspirated, and cells were washed once with warm phosphate-buffered saline (PBS). Fixation was done by incubating cells with 4% formaldehyde in PBS. The concentration of DAPI used was 0.01 µg/mL. The GFP to DAPI ratio was calculated using *ImageJ* software from five different image fields, and results were analyzed in GraphPad Prism. The experiment was performed in an air-conditioned laboratory maintained at 23 °C. The initial temperature of the surface was 21.5 °C, and the temperature was 20.9 °C after 10 min. No significant change in temperature was observed.

**Fig. 6:**
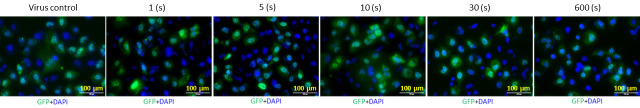
Experiment #1 UV-C. Representative data for fabric sample 4 with different UV-C exposure times. The DAPI (4′,6-diamidino-2-phenylindole)–stained nucleus is labeled in blue. GFP-expressing influenza viruses are shown in green.

Figure 5 shows the percentage of infected cells relative to the untreated virus control plotted against time of treatment. Error bars indicate the mean standard deviation of three replicate samples. Figure 6 shows the images for sample 4, and Appendix A.3 (Table 1) shows representative data of the GFP to DAPI ratio. The results do not indicate inactivation of the virus even after 10 min of exposure (600 s). As per Eq. (1) and as explained in Sec. 3.3, the exposure time required was 1.07 s for disinfecting H1N1 virus. One reason for the negative results could be that the virus was not exposed to sufficient UV dose for inactivation. This could be because (1) the UV dose assumed for the virus susceptibility to UV was too small; (2) there was insufficient UV intensity on the incident surface; or (3) there was less reflectivity from the internal walls within the test setup. According to Kowalski *et al*. [32], in an enclosed duct with UV radiation, a fraction of the incident UV intensity on its wall surface is reflected; *i.e.*, if the UV reflectivity of a wall material is 75%, then 75% of the UV intensity that falls onto the surface will be reflected back into the enclosed space [32]. These results were used to eliminate the concepts of an open configuration, and a closed-configuration design was chosen.

#### 3.3.2 Viricidal Efficacy of UV-C Disinfection Device (Closed Configuration)

**Fig. 7:**
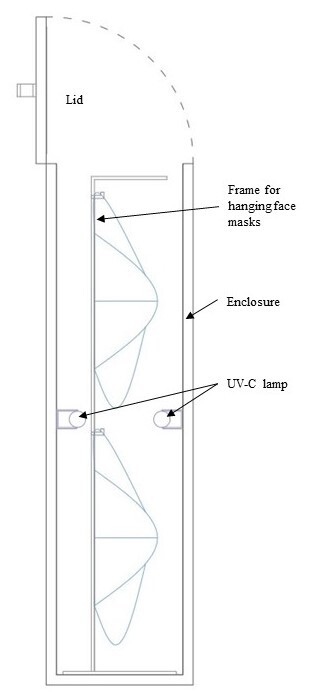

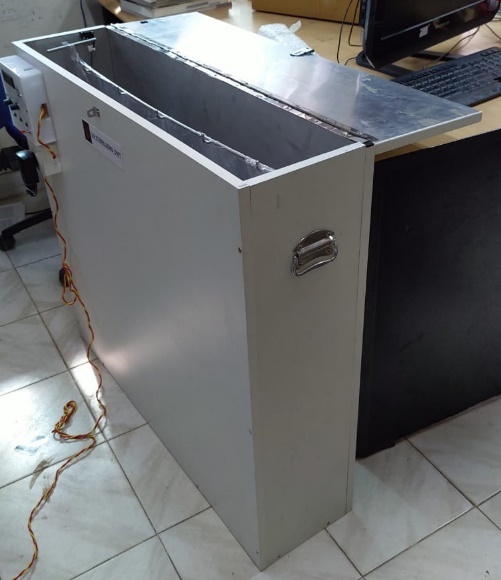
Cross section of UV-C disinfection product design (closed configuration) with dimensions L × W × D: 91 cm × 81 cm × 23 cm, and lamp length 43.8 cm (left), and photograph of the device (right).

Considering evidence from experiment #1, a closed-configuration UV-C disinfection device was designed and prototyped for testing for its use in viricidal efficacy evaluations. The product dimensions were (91 × 81 × 23) cm (length × width × depth). This prototype had internal walls lined with a 0.1 mm thick aluminum sheet, which has higher reflectivity for UV-C relative to the powder-coated sheet metal in the open configuration to ensure a more sufficient UV dose could be maintained. Two Osram Puritec 15 W UV-C lamps of length 43.8 cm were used in this device instead of one as in experiment #1 to increase the UV dose. A removable frame was provided with hooks for hanging face masks, N95 respirators, and PPEs (see Fig. 7).

##### 3.3.2.1 Viricidal Efficacy on PPE Fabrics

**Fig. 8:**
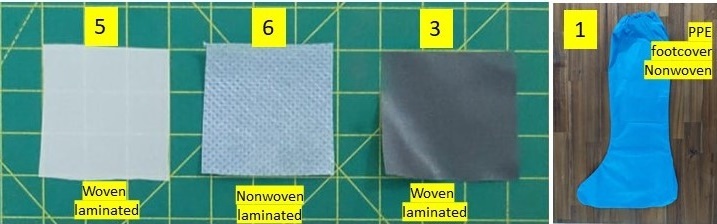
The four PPE samples used for viricidal efficacy experiments #2–4 UV-C with the closed-configuration design shown in Fig. 7.

Three PPE coverall fabrics samples—nonwoven laminated (1), woven laminated (2)—and a PPE foot cover made of nonwoven fabric (sample 1 of experiment #1 UV-C) were used as test samples (see Fig. 8). Three different disinfection methods were carried out on these samples to compare the viricidal efficacies.

As this work was carried out during lockdown in India due to COVID-19 pandemic, the availability of laboratories and research staff was limited; therefore, experiments #2–7 were conducted in a different laboratory than experiment #1. The hemagglutination assay (HA) test was used instead of A549 cells with GFP/DAPI method in the following experiments. The hemagglutinin protein on the surface of influenza virus particles is capable of binding to N-acetylneuraminic acid–containing proteins on avian and mammalian erythrocytes. When combined, if the influenza virus is present in a high enough concentration, there is an agglutination reaction, and the erythrocytes link together to form a diffuse lattice. The HA is a classic diagnostic test used to screen embryo tissue homogenate supernatant, cell culture fluids, or amniotic-allantoic fluid (AAF) harvested from embryonated chicken eggs. The test is to some extent quantitative because one hemagglutinating unit (HAU) is equal to approximately 10^5^ infectious viral particles . Live and inactivated viruses can be detected by the HA test. It is inexpensive and relatively simple to conduct [33]. Since the HA assay is a simple method and easy to conduct, it was used for experiments #2–7 because there were more test cases in these experiments. As the goal of conducting the following experiments was to ascertain the viricidal efficacy of the UV-C lamp and the corresponding exposure time and UV-C dose required to inactivate the virus, it was not essential to quantify the amount of live virus. The exposure time at which no live virus was detected was of significance to this experiment to arrive at the product design specifications of the UV-C disinfection device.

**Fig 9:**
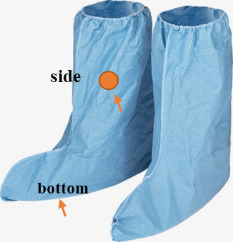
Two areas of the foot cover were tested: side and bottom.

In terms of specificity and the ability to detect reduction in influenza virus titers, the HA test can be used as an alternative to the fluorescence assay done in experiment #1. So, in both cases, the results would be similar, differing only in the way of interpretation. The influenza virus HA test can be reliably used for detecting differences in virus titer in samples. The test protocols of these are explained below:

• Experiment #2 UV-C: 2 cm × 2 cm areas of the fabric samples and foot cover were clipped to the frame and kept inside the middle of the UV closed-configuration device shown in Fig. 7 (13.8 cm from the right lamp and 6 cm from the left lamp). Then, 128 HAU/50 µL (approximately 1.2 × 10^7^ PFU) aliquots of A/WSN/1933 (H1N1) virus present in 20 µL were spotted on these samples in triplicate. For the foot cover, the shadow areas where UV radiation did not directly fall were chosen as spots for testing (Fig. 9). The samples were exposed to UV-C for time intervals of 10 s, 30 s, 60 s, and 10 min.

After UV-C exposure, the samples, including control samples, were washed with 1 mL of Dulbecco’s modified Eagle’s medium (DMEM) to extract the virus in order to add it to the test cells to check their infectivity after UV-C exposure. Next, a 50 µL volume of viral wash was added to a confluent monolayer of MDCK cells, which supported the replication of influenza viruses. Then, 96 well plates of 6.4 mm diameter were used, and 50 000 MDCK cells were seeded per well. The virus was incubated with the cells for 1 h to allow the viable virus to adhere to the cells, after which the viral inoculum was removed and washed with 1× PBS. Next, a 100 µL volume of DMEM media was added to the cells and incubated at 37 °C and 5% CO_2_ for 72 h. After 72 h, the HA assay was performed. Briefly, a 50 µL volume of supernatant with cells was added to 50 µL of Guinea pig red blood cells (RBCs) and incubated for 45 min at room temperature to check for virus presence. If the virus is viable when added to the cells, it will replicate to a higher titer and agglutinate the RBCs to form a lattice. In the absence of virus, or in case the virus was inactivated by UV-C or due to other factors, it will not be able to replicate in the cells or agglutinate the RBCs, and the latter will settle down and form a red dot in the bottom of the well.

The controls used in the experiment were the following: (1) One sample of 2 cm × 2 cm for each fabric type with the virus spotted on was kept without any exposure to UV-C as a virus control, and it was washed from the fabric samples and used to infect the cells as explained above (labeled UV0 in Table 4); (2) one cell control (C) sample was used; (3) an untreated virus control was kept on 96 well plate without any disinfection treatment and washed with the same volume of DMEM as for the fabric samples and used to infect the cell; (4) a virus control was kept on a 96 well plate and chemically disinfected immediately (V0); (5) a virus control was kept on a 96 well plate and chemically disinfected at time = 10 min (V10); and (6) a PBS control (only RBCs) was used.

The HA assay is not an accurate quantification method compared to the plaque assay.^3^3 The plaque assay is a standard method used to determine the virus titer or infectious dose [34]. In this experiment, the aim was not to quantify the live virus but to confirm the presence or absence of the virus after 72 h from infecting the cells and UV-C exposure. One HA unit corresponds to 10^4^ particles per milliliter in a standard condition [34]. Even if one infectious viral particle was intact, after 72 h from infecting the cells, it will multiply to a higher titer more than the minimum 1 HA unit, and the presence of the virus can be detected with the HA assay. The virus titer in a sample can be estimated by multiplying the dilution folds.

• Experiment #3 Chemical: In this experiment, the same samples were treated with a chemical disinfectant after spotting with 20 µL (128 HAU) of virus. These were disinfected with 50 µL of chemical disinfectant (80 volume percent ethanol, 1.45 volume percent glycerine, and 0.125 volume percent hydrogen peroxide). The chemical disinfectant was spread on the samples using a tip at the same spot where the viral suspension was added and immediately washed and used to infect the cells. The rest of the test method was the same as experiment #2 for the controls, collection of the virus, and detection methods.
• Experiment #4 Chemical + UV-C: Here, the samples were treated with chemical disinfectant followed by UV-C exposure for 10 min in the UV device. The procedures used for chemical disinfection were the same as in experiment #3, and the procedures used for HA and the controls were the same as in experiment #2.

**Fig. 10:**
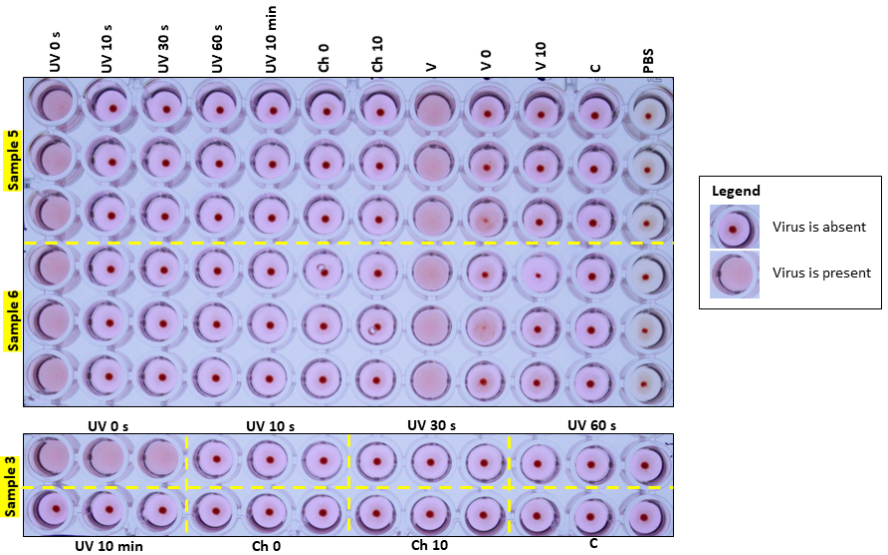
Experiments #2, 3, and 4: RBC agglutination image after 72 h from infecting the cells and UV-C exposure of PPE fabric samples numbered 5, 6, and 3. Legend: Same as in Table 4. Red dot indicates RBC agglutination and the virus is inactivated (the virus is absent). No dot indicates the virus was not inactivated (the virus is present).

**Fig. A.3.1:**
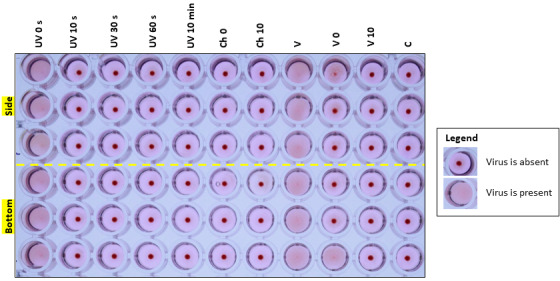
Experiments #2, 3, and 4: RBC agglutination image of PPE foot cover sample. Legend: Refer to Table 4 for column labels. Refer to Fig. 9 for positions of side and bottom sample areas. Red dot indicates RBC agglutination, where the virus is inactivated (the virus is absent). No dot indicates the virus was not inactivated (the virus is present).

Table 4 displays the test cases and results of experiments #2–4 for all triplicate samples. The experiment was performed in an air-conditioned laboratory maintained at 23 °C. The initial temperature of the surface was 23.5 °C, and the temperature was 20.9 °C after 10 min. No significant change in temperature was observed. Results from experiments #2–4 demonstrated the virus control (untreated virus sample) replicated in the MDCK cells and hemagglutinated the RBCs, forming a lattice. The initial dose of virus control was the same as the dose used in the test samples (1.2 × 10^7^ PFU/20 µL), which was treated the same way as the test sample, and the final dose of virus control used was 6.4 × 10^5^ PFU. The cloth and the foot cover samples showed viral replication when not disinfected by any method (*i.e.*, untreated fabric with UV-C exposure time = 0 s). After UV-C exposure, the virus did not show any replication activity at any of the time points tested. Even the samples from the shadowed area in the foot cover, which was not exposed directly to UV-C, did not show viral replication. This indicates that UV-C inactivated the virus even in shadowed areas. Chemical disinfection with and without UV-C exposure also disinfected the virus. Figure 10 and Fig. A.3.1 show the RBC agglutination images of PPE fabric samples and foot cover, respectively.

##### 3.3.2.2 Viricidal Efficacy on N95 Respirators

**Fig. 11:**
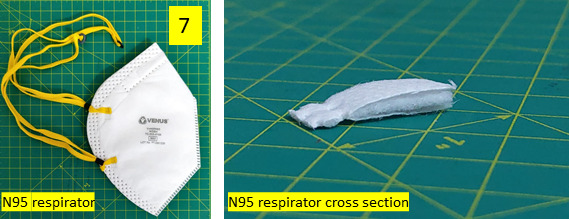
Cross section of N95 respirator sample.

Three different three-layered N95 respirators were used as test samples (see Fig. 11). Similar to the tests on the fabrics, three different disinfection cases were used for comparing the viricidal efficacies. The test protocols of these are explained below:

• Experiment #5 UV-C: Here, a 128 HAU/50 µL (approximately 1.2 × 10^7^ PFU) aliquot of A/WSN/1933(H1N1) virus was spotted on a 4 cm × 4 cm area of each respirator. The samples were UV-C treated in the UV-C closed-configuration device (see Fig. 7) for the time intervals of 10 s, 30 s, 60 s, and 10 min. The respirators were distributed in the UV-C device in a row with one respirator in the center and one each to its left and right sides. Separate samples were collected from the outer (*i.e.*, outward facing from the human face when worn), middle, and inner (*i.e.*, inward facing to the human face when worn) layers. The inner layer surface received the least UV-C exposure, as it was in a shadow area while placed in the UV device. The sandwiched middle layer had no direct exposure to UV-C, and it was tested after 10 min of UV-C exposure. The experiment was done in triplicate, *i.e.*, 3 replicates per treatment. The spots were cut and washed with 1 mL of DMEM, and a 50 µL volume of inoculum was added to MDCK cells. The experiment was carried out similarly to the experiment explained previously in Sec. 3.3.2.1 for experiment #2 UV-C. One sample of 4 cm × 4 cm area of each respirator was kept without any treatment as a virus control (UV0). The other controls used in the experiment were similar to experiment #2.
• Experiment #6 Chemical: In this experiment, the respirator samples were treated with a chemical disinfectant after spotting 20 µL (128 HAU) of virus. These were disinfected with 50 µL of chemical disinfectant. The chemical disinfectant used was the same as in Sec. 3.3.2.1 for experiment #4. The other test protocols were the same as in Sec. 3.3.2.2 for experiment #5 for collection and detection of the virus.
• Experiment #7 Chemical + UV-C: Here, the respirator samples were treated with chemical disinfectant followed by UV-C exposure for 10 min in the UV-C closed-configuration device. The other test protocols were the same as in Sec. 3.3.2.1 for experiment #4 for chemical disinfectant application and as Sec. 3.3.2.1 for experiment #2 and Sec. 3.3.2.2 for experiment #5 for exposure to UV-C and collection and detection of the virus.

Table 5 displays data for the test cases and the results of experiments #5–7 for all triplicate samples. The untreated virus control agglutinated the RBCs, indicating active virus without UV exposure. The virus was inactivated after 10 s exposure to UV for the outer, middle, and portions of the inner layers, as it did not show any replication as indicated by the absence of HA activity. However, the virus was only inactivated on shadowed areas of the inner layer after 60 s. Since the shadowed areas of the inner layer were not exposed directly to UV-C, it took more time to inactivate the virus. Chemical disinfection with and without UV-C exposure also inactivated the virus.

#### 3.3.3 Discussions of Viricidal Efficacy Tests for Experiments #1–7

The redesign of the open UV-C device after experiment #1 to a closed-configuration design to increase the UV dose by adding an additional UV lamp and UV-C reflective material was found to be effective based on data from experiments #2, 4, 5, and 7. This suggests that many open-configuration UV-C lamps in the market, such as handheld UV wands and UV-C room devices with UV lamps open to wide areas without specific targets, are not effective disinfectant devices for surface disinfection. The theoretical calculation of exposure time was 1.07 s for the H1N1 virus. While we did not perform tests at 1.07 s, the results of the tests on PPE fabric samples from experiments #2 and 4 showed no viral replication at the lowest time stamp where measurements were taken, which was 10 s. Viricidal efficacy tests from 10 s to below 1.07 s would be needed to verify the theoretical calculation.

In the closed configuration with UV-C reflective material lining, the shadowed areas where UV-C radiation did not directly fall onto the viral spots were also disinfected at the lowest time stamp of 10 s. This is observable in results from experiment #2, where one of the samples was a PPE foot cover. The foot cover is a large fabric piece that had folds and creases while it hung in the UV-C closed-configuration product’s frame. Despite this, the virus was inactivated immediately at 10 s and remained inactivated throughout the time study (see Table 4). This is also observable in experiment #5, where the virus on the inner layer of N95 respirators was inactivated. The inner layer had an inward fold, causing a shadowed area. In this case, the test samples were disinfected in 60 s for triplicates (see Table 5). One sample each inactivated the virus at 10 s and 30 s exposure times. This means complete disinfection is feasible for shadowed areas by increasing the exposure time of UV-C. Based on the data in Table 4, the use of UV-C disinfection for making PPE coveralls reusable is a potential practice during a time of shortage of PPEs during pandemic outbreaks, and similarly so for N95 respirators (based on data in Table 5), although respirator reusability requires further evaluations, such as filter and flow efficiency and fit, to determine if they are reusable.

From experiments #3, 4, 6, and 7 involving chemical disinfection, the results show that chemical disinfection also guarantees inactivation of the virus. However, the time and effort needed for this mode of disinfection are more than for UV-C disinfection. This is a wet process, whereas UV-C is a dry process, and the fabric needs to be dried in the former after the disinfection treatment. Another disadvantage of chemical disinfectants is that material handling of disinfectants by users requires training and skill, and there is safety concern during storage or for accidental consumption by humans. For UV-C, though its exposure to humans is harmful [35], the safety of users can be achieved through the design of the device itself, including an auto shut-off and well-sealed design options. The convenience of a dry process and less disinfection time shows promise for UV-C disinfection in high-volume disinfection needs during a pandemic outbreak like COVID-19.

In this work, we used enveloped RNA viruses in place of SARS-CoV-2. The results helped to confirm the two UV-C product design configurations, geometries, materials, the design calculations for UV doses required for a virus, and the lamp selections and placements. It is therefore useful to compare the two test configurations of the UV-C setup. Table 6 shows all the different experimental protocols used in experiments #1–7.

**Table 4:** UV-C viricidal efficacy results for experiments #2–4.^a^

	Disinfection Methods						Controls					
	Experiment #2				Experiment #3	Experiment #4						
Test Samples (in Triplicate)	UV 10 s	UV 30 s	UV 60 s	UV 10 min	Chemical Disinf. (Ch 0)	Chemical Disinf. + UV 10 min (Ch 10)	Cell Control (C)	Untreated Fabric (UV 0 s)	Untreated Virus Control (V)	Virus Only Chemically Disinfected (V0)	Virus Only Chemically Disinfected at Time 10 min (V10)	Buffer Control, Only RBCs (PBS)
Sample 5; woven, laminated	✓	✓	✓	✓	✓	✓	✓	✘	✘	✓	✓	✓
	✓	✓	✓	✓	✓	✓	✓	✘	✘	✓	✓	✓
	✓	✓	✓	✓	✓	✓	✓	✘	✘	✘	✓	✓
Sample 6; nonwoven, laminated	✓	✓	✓	✓	✓	✓	✓	✘	✘	✓	✓	✓
	✓	✓	✓	✓	✓	✓	✓	✘	✘	✘	✓	✓
	✓	✓	✓	✓	✓	✓	✓	✘	✘	✓	✓	✓
Sample 3; woven, laminated	✓	✓	✓	✓	✓	✓	✓	✘	✘	✓	✓	✓
	✓	✓	✓	✓	✓	✓	✓	✘	✘	✓	✓	✓
	✓	✓	✓	✓	✓	✓	✓	✘	✘	✓	✓	✓
PPE foot cover of sample 1 nonwoven fabric; side (shadow area)	✓	✓	✓	✓	✓	✓	✓	✘	✘	✘	✓	✓
	✓	✓	✓	✓	✓	✓	✓	✘	✘	✓	✓	✓
	✓	✓	✓	✓	✓	✓	✓	✘	✘	✓	✓	✓
PPE foot cover of sample 1 nonwoven fabric; bottom (shadow area)	✓	✓	✓	✓	✓	✓	✓	✘	✘	✓		
	✓	✓	✓	✓	✓	✓	✓	✘	✘	✓		
	✓	✓	✓	✓	✓	✓	✓	✘	✘	✘		

^a^ Legend: C: Cell control; UV 0: untreated fabric sample with virus spotted on and no UV exposure; UV 30: UV exposure for 30 s in UV device; UV 60: UV exposure for 60 s in UV device; UV 10 min: UV exposure for 10 min in UV device; Ch 0: chemically disinfected; Ch 10: chemically disinfected + UV-C exposure for 10 min in UV device; V: untreated virus control in 96 well plate; V0: virus only chemically disinfected; V10: virus only chemically disinfected at time 10 min; PBS: phosphate-buffered saline control (only RBCs); NA: test not carried out. ✘: virus was not inactivated (the virus is present); ✓: virus was inactivated (the virus is absent).

### 3.4 UV-C Reflectivity Test

In a closed-configuration UV-C device, the selection of internal wall material with a desired UV-C reflectivity is a crucial design aspect for achieving the required UV dose within a device. Four UV-C devices of closed configuration with four different internal material finishes were tested for their UV-C reflectivity. An Ocean Optics USB4000 spectrometer was used to measure the percent reflection at 45° and 90° from the illumination source for three internal walls to evaluate the UV dose at the surface for disinfection at these angles. Table 7 shows the results. From Table 7, the UV-C reflectivity of a UV-C device with polished aluminum internal finish is 35-15, which implies 35% of UV-C from the lamp source

**Table 5:** Viricidal efficacy results for experiments #5–7.^a^

	Disinfection Methods						Controls					
	Experiment #5				Experiment #6	Experiment #7						
Test Samples (in Triplicate)	UV 10 s	UV 30 s	UV 60 s	UV 10 min	Chemical Disinf. (Ch 0)	Chemical Disinf. + UV 10 min (Ch 10)	Cell Control (C)	Untreated Fabric (UV 0 s)	Untreated Virus Control (V)	Virus Only Chemically Disinfected (V0)	Virus Only Chemically Disinfected at Time 10 min (V10)	Buffer Control, Only RBCs (PBS)
N95 respirator—outer layer	✓	✓	✓	✓	✓	✓	✓	✘	✘	✓	✓	✓
	✓	✓	✓	✓	✓	✓	✓	✘	✘	✓	✓	✓
	✓	✓	✓	✓	✓	✓	✓	✘	✘	✓	✓	✓
N95 respirator—middle layer	NA	✓	NA	✓	NA	NA	NA	✘	✘	✓	✓	✓
	NA	✓	NA	✓	NA	NA	NA	✘	✘	✓	✓	✓
	NA	NA	NA	NA	NA	NA	NA	✘	✘	✓	✓	✓
N95 respirator—inner layer	✘	✘	✓	✓	✓	✓	✓	✘	✘	✓	✓	✓
	✓	✓	✓	✓	✓	✓	✓	✘	✘	✓	✓	✓
	✘	✘	✓	✓	✓	✓	✓	✘	✘	✓	✓	✓

^a^ Legend: Same as in Table 4.

**Fig. A.4.1:**
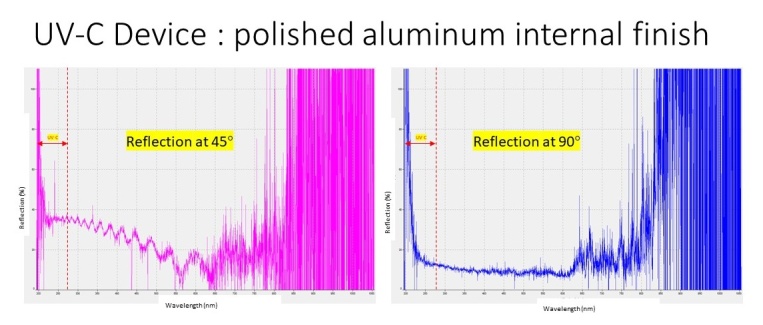
Reflection percentage spectrum of surface #1 in closed-configuration UV-C device with polished aluminum internal finish at 45° and 90°. The *y* axis is the reflection percentage. The *x* axis is wavelength in nanometers. UV-C spectrum is marked in the figure.

was detected at 45°, and 15% of UV-C was detected at 90° for surface #1. Figure A.4.1 shows the UV-C intensity spectrum and percentage reflectivity for the UV-C device with polished aluminum internal finish at 45° and 90°. The percent reflection values at other angles were not measured. Similarly, for a UV-C device with rough aluminum internal finish, 5% of UV-C from the lamp source was detected at both 45° and 90°. This result could be used to determine the UV-C exposure time while operating the particular UV-C device or to make design decisions such as to place more lamps, change the internal material, or modify object placement, and to revise the product design.

### 3.5 UV-C Product Certification

Before their release to market, UV devices must be tested to report the essential disinfection properties, in particular, UV-C (or other type) intensity, wavelength, and dose at boundary points of the product to describe the product performance. Product safety for human use must also be checked for UV radiation leaks through shutters or joints and any chances of accidental human exposure. The closed-configuration UV-C device was tested for these requirements.

#### 3.5.1 UV-C Properties

The UV-C device was tested to detect the UV-C properties (intensity, wavelength, and dose) using an Ocean Optics USB4000 spectrometer at spots where the object to be disinfected would be placed. This would verify the minimum exposure time necessary for the UV-C device to disinfect objects placed at the farthest points from the lamp source, provided the required UV-C dose for a virus is known. If the detected

**Table 6:** Experimental methods carried out for experiments #1–7 for viricidal efficacy.

	Expt. #1 UV-C	Expt. #2 UV-C	Expt. #3 Chemical	Expt. #4 Chemical + UV-C	Expt. #5 UV-C	Expt. #6 Chemical	Expt. #7 Chemical + UV-C
Disinfection device	Device 1	Device 2	Chemical disinfectant	Chemical disinfectant + Device 2	Device 2	Chemical disinfectant	Chemical disinfectant + Device 2
Test samples	PPE coverall fabric, N95 respirator	N95 respirator	N95 respirator	N95 respirator	PPE coverall fabric, foot cover	PPE coverall fabric, foot cover	PPE coverall fabric, foot cover
Sample sterilization method	UV 30 min, antibiotics in media	No sample sterilization was carried out; instead, antibiotics were added in the media					
Virus and cells	Cal/09 GFP; A549 cells	Influenza A/WSN/1933 (H1N1) virus					
Application method	50 µL Cal/09 GFP spotted on fabric samples	20 µL of influenza A/WSN/1933 (H1N1) virus spotted on fabric samples					
Disinfection step	UV-C at 15 cm distance for time intervals: 1 s, 5 s, 10 s, 30 s, 10 min	UV-C for time intervals: 10 s, 30 s, 60 s, 10 min	50 µL chemical disinfectant	50 µL chemical disinfectant + UV-C 10 min	UV-C for time intervals: 10 s, 30 s, 60 s, 10 min	50 µL chemical disinfectant	50 µL chemical disinfectant + UV-C 10 min
Infectivity step	100 µL inoculum added to A549 cells	50 µL inoculum added to a monolayer of MDCK cells					
Detection method	Effects on virus titer analyzed by fluorescence microscopy	Effects on virus replication analyzed by infectivity assay followed by detection using HA assay					
Control	Infected sample with no UV exposure	Infected sample with no UV exposure, untreated virus control, negative control sample					
Number of tests	Triplicate	Triplicates for each outer, middle, inner layer	Triplicates for outside, inside layers	Triplicates for outside, inside layers	Triplicates	Triplicates	Triplicates

UV-C dose is below the UV-C susceptibility of a virus, then the exposure time must be increased to achieve sufficient dose. The UV-C device (IISc UV-C device) provided a UV-C dose greater than 211 J/m^2^, which was more than the 100 J/m^2^ estimated using theoretical calculations. We carried out tests for 42 other UV-C disinfection products from different companies during the period of April to August 2020. Table 8 shows the UV-C properties of selected products among those tested. While testing different UV-C lamps from outside organizations, we noted that an open-configuration UV-C device with light-emitting diode (LED) lamps showed drastically reduced intensity after a short distance (>64 027 counts to 2.13 m and 44 000 counts at 3.05 m), whereas an open-configuration UV-C device with tubes had good intensity at greater distances (>64 027 counts from 0.61 m up to 3.35 m tested range).

#### 3.5.2 Product Safety and Operational Hazard

This procedure involved two aspects:

1. Determine any operational hazard due to UV-C exposure during placement or removal of objects for disinfection or due to leakage through the product enclosure—To prevent this, the closed-configuration device was equipped with a timer set, which would cut off the circuit to the UV-C lamp if the door was opened accidentally or intentionally during a disinfection cycle. An emergency stop button was also incorporated in the design in case of any other unexpected accident.
2. Determine any UV-C wavelength leak from the product enclosure—This was checked using an Ocean Optics USB4000 spectrometer by placing the probe at joints of openings and recording the values. No UV leak was detected for the closed-configuration device. The same test method was followed to test 42 UV-C disinfection devices received from different organizations. The most common UV-C leak detection was in the gaps between the lid/door opening. Leaks were also detected in UV-C devices that had transparent glass doors and at joints on the device.

**Table 7:** Percent reflection of UV-C on different materials at 45° and 90° incident angles.

Internal Finish of Closed-Configuration UV-C Device	Percent Reflection of UV-C at 45° and 90° Angles on Three Surfaces (Values Shown in Percentage at 45°–90°)		
	Surface #1	Surface #2	Surface #3
Polished aluminum	35–15	35–15	30–10
Mild steel	30–20	38–18	35–18
Rough aluminum	5–5	5–5	5–5
Powder-coated aluminum	38–18	40–15	38–20

**Table 8:** UV intensity and dose detected for different designs.

Designs	UV Wavelength near Lamp Source (nm)	Intensity (counts)	UV Wavelength at Farthest Point (nm)	Intensity (counts)	UV Dose (J/m^2^)
1. IISc UV-C device (closed-configuration) device	250–280	65 000	250–280	65 000	>211
2. UV-C device (closed configuration)	270–280	68 000	270–360	68 000	>211
3. UV-C device (closed configuration)	250–260	60 000	250–255	40 000	>76.9
4. UV-C device (closed configuration)	250–255	65 000	250–255	28 000	>0.5
5. UV-C device (closed configuration)	250–275	65 000	250–255	65 000	>0.5
6. UV-C device (closed configuration)	250–260	65 000	250–255	50 000	>0.5
7. UV-C handheld	250–290	68 000	250–270	68 000	>211
8. UV-C handheld device	250–290	65 000	250–260	65 000	>0.5
9. UV-C device tunnel configuration	250–270	65 000	250–260	65 000	>0.5
10. UV-C device for space disinfection	250–260 (0.61 m)	65 000	250–255 (3.35 m)	65 000	>0.5

**Table 9:** Summary of a recommended product design process for UV-C disinfection products.

Design Stages	Design Guidelines
Planning and clarification of task or feasibility	Identifying customer needs is key factor in this stage while designing any product for a user segment. User research must be carried out to determine the following key design aspects: **1. Location/building use type where UV-C disinfection product would be used**—hospitals, home, public buildings, institutions. **2. Objects to disinfect**—general purpose (personal gadgets, keys, laptops, packaging materials); special purpose (PPE, face masks, gloves, surgical tools). **3. Target unit cost**—How much a potential customer would pay for a unit. Outcome of the user research determines the design requirements, which influence geometry, capacity, and material selection (lamp and enclosure design), and cost in the later stages. For example, in this work, the requirement was PPE disinfection for noncritical care use at public buildings.
Conceptual design	In the concept design phase, the design requirements of a product are identified, and alternative design concepts are generated and evaluated. Form, function, features, and components of the product would take shape at this stage. The parameters below are important at this phase in the design of a UV-C disinfection device: **1. Product geometry and capacity** The customer preferences from the user research and design requirements should lead the selection of overall product dimensions, capacity, and geometry (form). Product dimensions for a certain capacity (volume) can be arrived at by considering the following three aspects simultaneously: • object sizes intended to be disinfected, • space constraints derived from where the end user will place the product, and • size constraints posed by the selected UV-C lamp. Product geometry alternatives can be generated from the above considerations. **2. Selecting the UV-C lamp** For selecting a UV-C lamp, the following design considerations must be followed: • If a particular virus is targeted for disinfection (as in the case for COVID-19), identify the UV wavelength and D90 value (the minimum UV dose required to eliminate the virus by 90%) from scientific literature [27]. • Identify a UV-C lamp available in the market that has the required wavelength. • If time is one of the critical design requirements as identified by user needs: ○The target disinfection cycle time must be taken as the exposure time. This determines the intensity of lamps (wattage) and the number of lamps required to achieve the necessary UV-C dose for that duration, as in Eq. (1). ○ If time is not a constraint, choose a UV-C lamp as per availability and size constraints of the product and arrive at the exposure time. **3. Material selection** Enclosure design involves material selection of internal and external surfaces, and the opening. It is influenced by: • target unit cost of the product, • product size and geometry, and • target exposure time if design requirements demand it, in which case the internal material should be chosen as one that is a highly reflective material at the UV wavelength being used. Alternative materials may be considered at the concept design phase. Selection can be based on experimental measurements as in Table 7 or how well it meets the other design requirements. Many UV disinfection products available on electronic-commerce sites currently use a variety of enclosure material palettes, including sheet metal, plastic, and fabric (see Fig. 11). Due to lockdowns caused by the COVID-19 pandemic, or future constraints, the selection must be determined by availability of manufacturing facilities, existing product lines, production facilities, and the need to repurpose existing product supply chains, *etc*., if those are additional design constraints.
Embodiment design	In the embodiment design phase, the selected concept would take the form of more definitive size, geometry, and material specifications that meet the design requirements. In the UV disinfection product design, more details of form, sizes, clearances, enclosure design, control and electrical design, and safety features would be decided at this phase. Requirements derived from safety parameters, such as UV leaks, and design requirements, such as UV dose, ergonomics, and access to manufacturing facilities, can influence the product size, dimensions, and selection of materials and components. **1. Enclosure design** During this phase, the form and sizing would be worked out. Spatial constraints, if any, would be identified, like a UV-C lamp’s dimensions, clearances, and maintenance needs (UV-C lamp replacement). **2. Object placement trays and nesting** The design of trays for placing the object such that the UV-C shadow areas are minimized is carried out at this stage. The tray design must also consider the nesting needs of the object to be disinfected. **3. UV lamp placement and UV dose calculation** UV-C lamp placement is a key design decision, and the following aspects must be considered: • optimal orientation for a wider cone of radiation and • UV-C shadow area identification while objects are placed. If shadow areas present: • Decide to place additional lamp(s) to cover shadow areas. • Reorient the lamp orientation to cover the object. • Increase exposure time to compensate for shadow areas. • Choose a higher UV reflective material for the interiors of the enclosure. • Position the lamp so that a user is not exposed to UV-C while placing and removing objects. • Express the UV dose in millijoules per square centimeter (mJ/cm^2^) as calculated in Eq. (1). The longer time a microbe is exposed to UV radiation, the more exposure (total dose) it will receive [26]. **4. Control and electrical design** The control design and electrical design scheme take shape at this stage. Factors to be considered are: • exposure time, which is ideally preset to ensure the determined minimum exposure time (arrived at experimentally or theoretically) for each disinfection cycle; • indications when a cycle is ongoing and completed; • indication if the UV lamp is not working; • safety interlocks to cut off the UV lamp upon accidental/intentional opening of lid during a disinfection cycle; • general safety to ensure no electrical shock; and • emergency stop button.
Detail design	The detail design phase includes complete specification of the geometry, materials, and tolerances of all parts in the product and the identification of standard parts to be purchased from suppliers. Documentation of the product by drawings and computer-aided design (CAD) files describing each part would also be created in this stage [36]. Any critical issues pending decision making from the earlier stages are finalized in this phase, *e.g.*, material selection, selection of standard parts, production cost, *etc*. **1. Enclosure design** • Detailed drawings are made for how the materials (outer and inner, if different) are fixed for the enclosure design. • Joint detailing of all edges is set to prevent UV leakage. • Detailed drawings are made of lid attachment to enclosure and covering of gaps to prevent UV leaks. • Drawings are made of parts and standard parts, and bill of materials (BOM) and quality and testing documents are created. **2. Control and electrical design** • Circuit schematic drawings, wiring and harness drawings, printed circuit board (PCB) layouts, electrical and electronic BOM, and quality and testing documents are generated here.
Design for manufacture	At this stage, a prototype may be built prior to starting pilot production. Both the prototype and, if using batch production, selected products from a lot can be tested for product quality and functionality. For a UV-C disinfection product, improving the disinfection efficiency and product quality, eliminating UV leaks, minimizing production costs, and meeting product certifications are the key considerations at this stage. Two tests may be carried out at this stage: **1. Functional testing** • Test the viricidal efficacy of the product on the virus (prototype test and batch test). A laboratory test may be carried out as described in Sec. 3.3 or by any approved test methods for UV disinfection products that are available at the time of development. • Test the disinfection endurance cycle. A test may be carried out to determine how many cycles the object intended to be disinfected can withstand without deterioration or damage (similar to that described in Sec. 3.2). **2. Product safety testing** • Test UV leak detection (as described in Sec. 3.4.2). • Test design safety for user against UV exposure by user trials and observation of actual use.

## 4 Recommended Product Design Process for Designing UV-C Disinfection Devices

We presented the product development process for a UV-C disinfection device during the COVID-19 pandemic era, and the tests performed to evaluate each design stage. Designing a UV-C disinfection device involves design decision making that influences how well the final design meets the intended functions. This article presented the lessons learned and results of the product development process and the experiments performed to evaluate product performance; the survey of 99 UV-C disinfection products in the market; and the laboratory evaluations of 42 different UV-C disinfection prototypes, which helped to arrive at the important design aspects specific to designing UV-C disinfection devices for face masks and PPE. While the design of a UV-C disinfection device can be carried out following any generic product design process, the design process followed in this work [6, 7] had the following design stages: planning and clarification of task or feasibility; conceptual design; embodiment design; detail design; and design for manufacture. The key design aspects specific to a UV-C disinfection device at each stage of the design process are summarized in Table 9. The experiments presented in this paper are suggested as potential test methods to evaluate the efficacy of UV-C disinfection devices at each of the relevant design stages of a product. This article offers a recommended product design process for designing UV-C disinfection devices, design guidelines, and test methods (Table 9).

## 5 Conclusions

Designing new products for tackling the COVID-19 pandemic has been a challenge for individuals, organizations, and nations. UV-C disinfection offers advantages over chemical disinfectants as a noncontact disinfection method where surface contamination is one of the sources for transmission of the biological agents that cause COVID-19. The results demonstrate how a UV-C product configuration can affect the viricidal efficacy of a UV-C disinfection unit, showing promise for closed-configuration UV-C disinfection devices, which improve virus inactivation in very short time periods. The open-configuration UV-C device could not achieve virus inactivation after 10 min of continuous UV-C exposure. Chemical disinfectants were also shown to inactivate a virus load on PPE fabrics and N95 respirators. This work establishes the potential of UV-C as a dry disinfection method with which to disinfect the H1N1 virus on PPE fabrics and N95 respirators to enable their reuse, provided the design of the UV-C disinfection device can ensure sufficient UV dose on the object surface. While the virus load was inactivated on the N95 respirators investigated here, their reuse evaluation requires further testing for flow, filterability, and fit after UV-C exposure.

The test methods presented in this work could be adapted for UV-C disinfection of coronaviruses to achieve similar outcomes. As standards and test methods are not available to specifically certify a UV-C disinfection product for their use in disinfection, the test methods and approach presented and summarized in Table 9 are useful for designers as guidelines to confirm a product’s performance. Out of the 99 UV-C products surveyed in this work, 74 were designed as a general-purpose disinfection product but with little or no indication of efficacy. Therefore, there is need to validate the viricidal efficacy of UV-C and its effects on surfaces of daily-use objects such as food packets, plastics, other packaging materials, and medicines. This work performed an endurance test on PPE fabric samples, which are permeable to UV-C. While the fabric tensile strength was not assessed, the work describes an all-around study on designing and assessing a UV disinfection product. The product design guidelines and approach recommended in Table 9 will provide benefit to product designers in designing UV-C–based disinfectant products to achieve the minimum exposure duration time, lamp selection, enclosure design, and material selection. This work will also benefit researchers working on developing standards for UV-C disinfectant product design and efficacy.

## Figures and Tables

**Fig. A.1.1:**
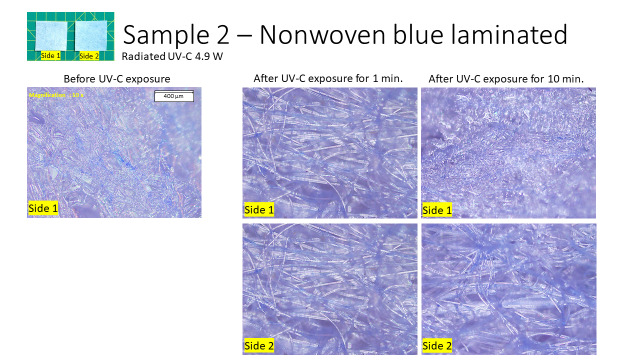
Endurance test #1 UV-C, sample 2: PPE coverall fabric (nonwoven, laminated) showing no changes as examined using an Olympus BX53M upright light microscope.

**Fig. A.1.2:**
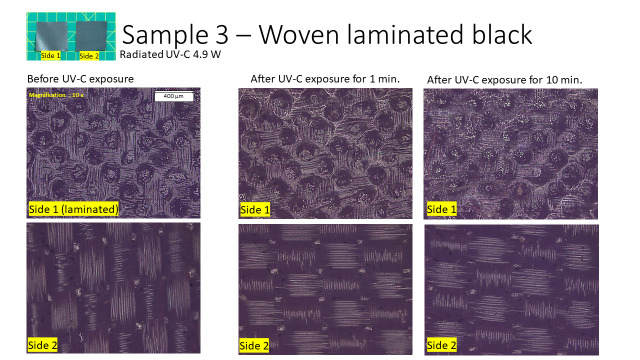
Endurance test #1 UV-C, sample 3: PPE coverall fabric (woven, laminated) showing no changes as examined using an Olympus BX53M upright light microscope.

**Fig. A.2.2:**
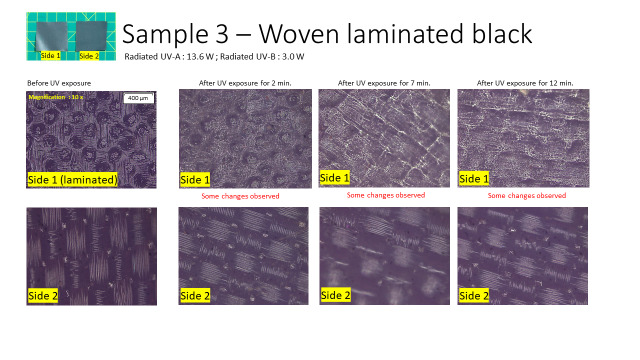
Endurance test #2 UV, sample 3: PPE coverall fabric (woven, laminated) showing burn-like changes as examined using an Olympus BX53M light microscope.

**Fig.A.2.3:**
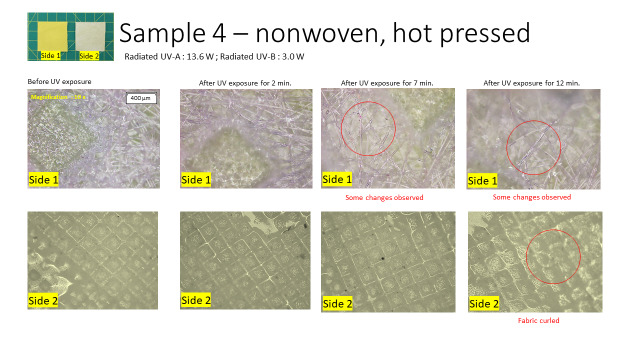
Endurance test #2 UV, sample 4: PPE apron fabric (nonwoven, hot-pressed) showing fiber thinning and burn-like changes as examined using an Olympus BX53M light microscope.

**Fig. A.3.2:**
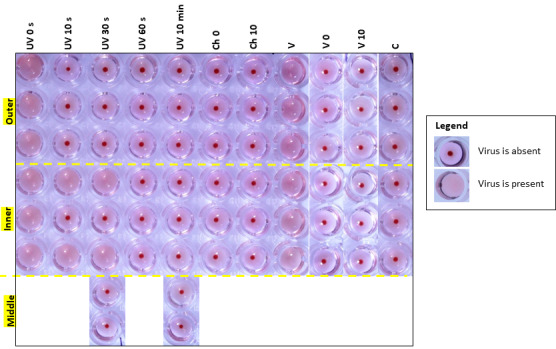
Experiments #5, 6, and 7: RBC agglutination image of N95 respirator. Legend: Refer to Table 5 in the article text for the legend. On the *y* axis: “Outer” refers to outer layer of respirator, “Inner” refers to innermost layer of respirator, and “Middle” refers to middle sandwiched layer of the respirator. The red dot indicates RBC agglutination, where the virus is inactivated (the virus is absent). No dot indicates the virus was not inactivated (the virus is present).

## 6 Appendix

### A.3 Viricidal Efficacy of UV-C on PPE Fabrics and N95 Respirators

**Table A.3.1:** GFP to DAPI ratio for experiment #1 UV-C. Representative data for sample 4.

Fabric 4	DAPI	GFP	Ratio
Virus control	52	32	0.62
1 s	58	32	0.55
5 s	72	43	0.60
10 s	65	40	0.62
30 s	52	26	0.50
600 s (10 min)	51	20	0.39
